# Pharmacological Investigations in Glia Culture Model of Inflammation

**DOI:** 10.3389/fncel.2021.805755

**Published:** 2021-12-16

**Authors:** Fatme Seval Ismail, Franco Corvace, Pedro M. Faustmann, Timo Jendrik Faustmann

**Affiliations:** ^1^Department of Neurology, University Hospital Knappschaftskrankenhaus Bochum, Ruhr University Bochum, Bochum, Germany; ^2^Department of Neuroanatomy and Molecular Brain Research, Ruhr University Bochum, Bochum, Germany; ^3^Department of Psychiatry and Psychotherapy, Medical Faculty, Heinrich Heine University Düsseldorf, Düsseldorf, Germany

**Keywords:** astrocyte-microglia co-culture model, M5/M30 conditions, inflammation, pharmacology, immunomodulatory drugs, psychotropic drugs, antiepileptic drugs (AEDs)

## Abstract

Astrocytes and microglia are the main cell population besides neurons in the central nervous system (CNS). Astrocytes support the neuronal network *via* maintenance of transmitter and ion homeostasis. They are part of the tripartite synapse, composed of pre- and postsynaptic neurons and perisynaptic astrocytic processes as a functional unit. There is an increasing evidence that astroglia are involved in the pathophysiology of CNS disorders such as epilepsy, autoimmune CNS diseases or neuropsychiatric disorders, especially with regard to glia-mediated inflammation. In addition to astrocytes, investigations on microglial cells, the main immune cells of the CNS, offer a whole network approach leading to better understanding of non-neuronal cells and their pathological role in CNS diseases and treatment. An *in vitro* astrocyte-microglia co-culture model of inflammation was developed by Faustmann et al. (2003), which allows to study the endogenous inflammatory reaction and the cytokine expression under drugs in a differentiated manner. Commonly used antiepileptic drugs (e.g., levetiracetam, valproic acid, carbamazepine, phenytoin, and gabapentin), immunomodulatory drugs (e.g., dexamethasone and interferon-beta), hormones and psychotropic drugs (e.g., venlafaxine) were already investigated, contributing to better understanding mechanisms of actions of CNS drugs and their pro- or anti-inflammatory properties concerning glial cells. Furthermore, the effects of drugs on glial cell viability, proliferation and astrocytic network were demonstrated. The *in vitro* astrocyte-microglia co-culture model of inflammation proved to be suitable as unique *in vitro* model for pharmacological investigations on astrocytes and microglia with future potential (e.g., cancer drugs, antidementia drugs, and toxicologic studies).

## Introduction

Astrocytes and microglia are the main cell population besides neurons in the central nervous system (CNS). Astrocytes represent the largest glia cell population. They are involved in the formation of the blood–brain barrier, support of the ion, water and neurotransmitter homeostasis as well as regulation of neuronal synaptogenesis (Giovannoni and Quintana, 2020). They are part of the tripartite synapse, which includes pre- and postsynaptic neurons and perisynaptic astrocytic processes as a functional unit (Araque et al., 1999; Möller et al., 2007). Importantly, astrocytes can form a syncytium by connecting individual cells to a large network using connexin 43 (Cx43), the main gap junctional protein (Reuss and Unsicker, 1998). Microglia, another type of glia cells, are the main immune cells of the CNS. They respond to changes of brain homeostasis under neuroinflammatory conditions with proliferation, activation and release of inflammatory mediators (Gehrmann et al., 1995; Voet et al., 2019).

Moreover, astrocytes and microglia play an important role in neurological diseases such as multiple sclerosis (MS) (Perriot et al., 2018; van der Poel et al., 2019; Wheeler and Quintana, 2019; Cignarella et al., 2020) and epilepsy (Coulter and Steinhäuser, 2015; Çavdar et al., 2019; Fu et al., 2021), but also in psychiatric diseases such as bipolar disorder and schizophrenia (Réus et al., 2015; Peng et al., 2016; Petrasch-Parwez et al., 2020). There is an increasing evidence that glia-mediated neuroinflammation is involved in the pathomechanism of these diseases (Vezzani et al., 2008; Réus et al., 2015; Perriot et al., 2018; Voet et al., 2019).

The involvement of astrocytes and microglia in neuropsychiatric disorders raises the question how these cells besides neurons might be responsive to current pharmacological treatments, especially with link to inflammation. Therefore, this focused mini review will summarize and discuss the major findings of pharmacological investigations in glia culture model of inflammation.

## *In vitro* Astrocyte-Microglia Co-Culture Model of Inflammation

**FIGURE 1:**
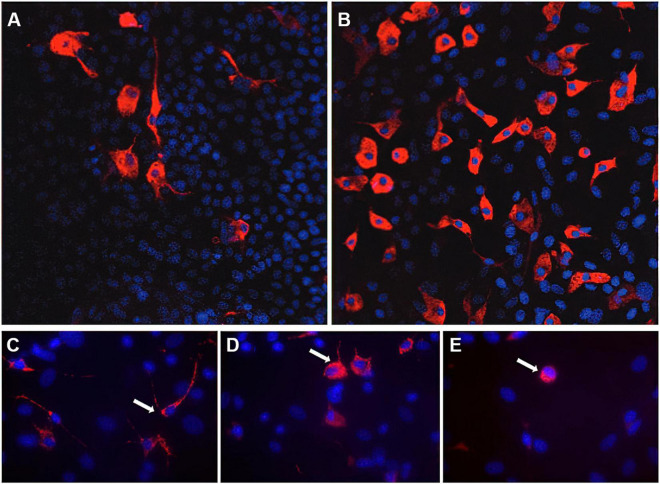
Immunocytochemistry of microglia morphology in M5 und M30 astrocyte-microglia co-cultures. Physiological M5 co-cultures containing 5% microglia (red) **(A)**. Pathological, inflammatory M30 co-cultures containing 30% microglia **(B)** (published by Dambach et al., 2014). Staining with the monoclonal antibody ED-1 allowed the classification of microglia (white arrows) as resting ramified **(C)**, intermediate **(D)** and activated rounded phagocytic **(E)** phenotype (published by Ismail et al., 2021). Nuclei (blue) were counterstained with DAPI to visualize the total glial cell number.

Since astrocytes and microglia are important players in healthy and diseased brain, Faustmann et al. (2003) developed an astrocyte-microglia co-culture model to study the physiological as well as pathological inflammatory states in the brain depending on the percentage and activation of microglia (Faustmann et al., 2003). Microglia play an important role in the maintenance of normal brain function (Ling and Wong, 1993). In health brain, they can be found as inactive ramified type (RRT), which represents the primary existing phenotype under physiological conditions (Ling and Wong, 1993; Faustmann et al., 2003; Kettenmann et al., 2011). Acute CNS lesions and pathologic changes lead to proliferation and activation of microglia, transitioning from the inactive ramified state *via* an intermediate form (INT) to the activated form (round phagocytic type, RPT) (Figure 1; Faustmann et al., 2003; Block et al., 2007; Kettenmann et al., 2011). The RRT has small cell bodies (5–10 μm) with a small perinuclear, cytoplasmic rim and thin branching processes longer than the diameter of the cell body (Figure 1C); a large cellular diameter, rare short processes, and several cytoplasmic vacuoles are typical for the activated RPT (Figure 1E); the INT is characterized by some thick pseudopodia longer than the diameter of the cell body and a perinuclear cytoplasmic rim with a few vesicles and vacuoles (Figure 1D; Faustmann et al., 2003). Under physiological conditions, the amount of microglia varies between 5 and 20% (Faustmann et al., 2003). The pathological activation of microglia occurs through several steps and is mediated by incoming stimuli such as ATP, adenosine, complement factors, cytokines, chemokines and changes in potassium concentration (Chang et al., 2009). As a result of activation, increased astrocyte mobility and a chemotactic gradient, microglia actively migrate to the pathological origin and exert curative or destructive effects depending on various factors (Walter and Neumann, 2009; Kettenmann et al., 2011). Under inflammatory conditions, activated microglia are able to produce neurotrophic factors, pro-inflammatory substances such as interleukin (IL)-6, tumor necrosis factor (TNF)-α, interferon (IFN)-γ as well as anti-inflammatory cytokines such as IL-10, transforming growth factor (TGF)-β (Ledeboer et al., 2000; Meeuwsen et al., 2003, 2005; Cartier et al., 2005). The effect exerted by microglia seems to depend in particular on stimulation and interaction with other molecules (Czeh et al., 2011; Biber et al., 2014; Chen and Trapp, 2016). The interactions between astrocytes and microglia have a crucial impact on neuroinflammation in the CNS and are not well understood. Murine *in vitro* astrocyte and microglia cultures are powerful tools to study molecular signaling pathways involved in neuroinflammation (Barbierato et al., 2013; Dambach et al., 2014; Facci et al., 2018; Ismail et al., 2021). In the astrocyte-microglia co-culture model developed by Faustmann et al., 2003, the physiological state is characterized by a microglia fraction of 5–10% (referred to as M5 co-culture) (Figure 1A), this fraction increases to 30–40% (M30 co-culture) (Figure 1B) under pathological, inflammatory conditions. In addition to the percentage, the M5- or M30-experimental paradigms also produce different microglia phenotypes. While the M5 co-culture contains predominantly resting ramified microglial cells (Figure 1A), more activated microglia are found in the M30 co-culture (Figure 1B; Faustmann et al., 2003; Hinkerohe et al., 2005). Moreover, a positive correlation of percent activated microglia with reduced astroglial Cx43 expression was demonstrated, suggesting a functional relationship between microglial activation and coupling efficiency in the astroglial network under *in vitro* conditions (Faustmann et al., 2003). Incubation of the M5 co-cultures with the pro-inflammatory cytokines further resulted in microglial activation itself (Hinkerohe et al., 2005). In contrast, incubation of pathological M30 co-cultures with TGF-ß1 resulted in a decrease of microglial activation including restoration of functional coupling *via* gap-junctions. Furthermore, IFN-ß prevented the effects of the pro-inflammatory cytokines TNF-α, IL-1β, and IFN-γ in M5 co-cultures (Hinkerohe et al., 2005). Since the presence of microglia under *in vivo* conditions is considered crucial for the extent of inflammation in various neurological diseases such as MS (Bogie et al., 2014; Giunti et al., 2014) or Alzheimer’s disease (Gold and El Khoury, 2015; Heppner et al., 2015; Malik et al., 2015; ElAli and Rivest, 2016), this simple but efficient co-culture model, compared to classical monocultures, allows the mimicking of inflammatory conditions in a defined *in vitro* assay, thus not only the activation of microglia, but also the response of astrocytes to this activation in terms of bidirectional interactions can be studied (Faustmann et al., 2003; Hinkerohe et al., 2005). Besides the obvious advantage of this established cell culture model over monocultures, which do not take the interactions between astrocytes and microglia into account, the advantage over other co-cultures must also be considered. Most astrocyte-microglia co-cultures are based on two primary cultures (astrocytes and microglia) cultivated together in different ratios, whereas in the system developed by Faustmann et al. (2003) a much more natural inflammation model is obtained by activation of microglia and concomitant proliferation (Bohatschek et al., 2001; Faustmann et al., 2003; Zhao et al., 2017). Of course, the limitation of the model to tricultures, which include neurons in addition to astrocytes and microglia, must also be considered (Goshi et al., 2020). Such a triculture model mimics neuroinflammatory responses correspondingly more accurately, but overall, due to its uniqueness and ease of reproducibility, the co-culture system established by Faustmann et al., 2003 is an excellent model for studying neuroinflammatory responses between astrocytes and microglia, cell-cell communication and their interaction on pharmaceuticals (Table 1; Hinkerohe et al., 2005, 2010, 2011; Haghikia et al., 2008; Vollmar et al., 2008; Stienen et al., 2011; Dambach et al., 2014; Moinfar et al., 2014; Ismail et al., 2021).

**TABLE 1:** Pharmacological investigations in astrocyte-microglia co-culture model of inflammation.

	M5 (physiological) co-cultures				M30 (pathological, inflammatory) co-cultures				
	Microglia phenotypes	Cytokine expression	Cx43 expression	Gap-junctional communication	Microglia phenotypes	Cytokine expression	Cx43 expression	Gap-junctional communication	References
Levetiracetam	–	TGF-β1 ↑	After IL-1β and LPS treatment Cx43 ↔	After IL-1β and LPS treatment ↔	–	IL-1β↓ TGF-β1 ↑	Cx43 ↑	↑	Haghikia et al., 2008; Stienen et al., 2011
Valproic acid	Activated microglia ↑	TGF-β1 ↔ TNF-α↔	Cx43 ↔	–	Activated microglia ↑	TGF-β1 ↔ TNF-α↔	Cx43 ↔	–	Dambach et al., 2014
Carbamazepine	Microglia ↔	TGF-β1 ↔ TNF-α↔	Cx43 ↔	–	Activated microglia ↓ Inactivated microglia ↑	TGF-β1 ↔ TNF-α↔	Cx43 ↔	–	Dambach et al., 2014
Phenytoin	Activated microglia ↑ Inactivated microglia ↓	TGF-β1 ↑* TNF-α↔	Cx43 ↔	–	Microglia ↔	TGF-β1 ↑ TNF-α↑	Cx43 ↔	–	Dambach et al., 2014
Gabapentin	Microglia ↔	TGF-β1 ↑* TNF-α undetectable	Cx43 ↔	–	Microglia ↔	TGF-β1 ↑* TNF-α↔	Cx43 ↔	–	Dambach et al., 2014
Dexamethasone	After LPS treatment Activated microglia↓	–	After LPS treatment Cx43 ↑	After LPS treatment ↑	Activated microglia ↓* Inactivated microglia ↑	–	Cx43 ↑	↑*	Hinkerohe et al., 2010, 2011
Interferon-β	After TNF-α, IL-1β, IFN-γ treatment Activated microglia↓	–	–	After TNF-α, IL-1β, IFN-γ treatment ↑	–	–	–	–	Hinkerohe et al., 2005
Venlafaxine	–	–	–	↔	Activated microglia ↓ Inactivated microglia↑	TGF-β↑ IL-6↓ IFN-γ↓ IL-10↔	–	↑*	Vollmar et al., 2008
Ammonia	Activated microglia ↑ Inactivated microglia ↓	–	Cx43 ↔	–	Activated microglia ↑ Inactivated microglia ↓	–	Cx43 ↑	–	Ismail et al., 2021

*Cx43, connexin 43; IFN-γ, interferon-γ; IL-1β, interleukin-1β; IL-6, interleukin-6; IL-10, interleukin-10; TGF-β1 transforming growth factor-β1; TNF-α, tumor necrosis factor-α; IFN-β, interferon-β; LPS, lipopolysaccharide.*

↑, increase; ↓, decrease; ↔, unchanged; –, not available; *, at higher concentration.

## Pharmacological Investigations

### Antiepileptic Drugs

Several studies provide evidence that astrocytes and microglia are involved in the pathophysiology of epilepsy (Bedner et al., 2015; Berger et al., 2019; Patel et al., 2019; Heuser et al., 2021). Clinical and experimental research showed that epileptic activity can be associated with inflammation due to increased levels of inflammatory mediators in the brain (e.g., IL-1β, TNF-α, and IL-6), which are produced by glia, neurons, endothelial cells of the blood–brain barrier and peripheral immune cells (Vezzani and Granata, 2005; Vezzani et al., 2011). Additional cellular mechanisms of inflammation such as reactive astrocytosis and activated microglia are well known (Vezzani and Granata, 2005; Vezzani et al., 2011). The anticonvulsant effect of immunomodulatory drugs with anti-inflammatory actions such as adrenocorticotropic hormone (ACTH) and steroids has been demonstrated (Brunson et al., 2002; Granata et al., 2003; Vezzani and Granata, 2005). However, the question remained whether available antiepileptic drugs (AEDs) may have anti-inflammatory effects. For this reason, we have investigated pro-and anti-inflammatory effects of conventional and second-generation AEDs in our astrocyte-microglia co-culture model of inflammation (Table 1). We showed a significant microglial activation in physiological M5 and inflammatory M30 co-cultures after concentration-dependent incubation with valproic acid (VPA) (Dambach et al., 2014). Incubation with gabapentin (GBT) induced no significant alterations in the microglial activation state. Phenytoin (PHE) led to increase of amount of activated microglia (RPT) in M5 co-cultures, whereas incubation of M30 co-cultures with PHE did not affect the microglial phenotypes (Dambach et al., 2014). In another study, sodium channel blockade with PHE significantly reduced the phagocytic activity of lipopolysaccharide (LPS)-activated microglia (Black et al., 2009). Furthermore, carbamazepine (CBZ) significantly reduced the amount of activated microglial cells in M30 co-cultures. This finding is consistent with anti-inflammatory properties of CBZ in rat models with regard to pain/hyperalgesia (Bianchi et al., 1995; Iwamoto et al., 2011). In addition, increased TGF-β1 and TNF-α cytokine levels were detected after incubation with PHE in M30 co-cultures. The other AEDs VPA, GBT, and CBZ did not alter the TNF-α cytokine expression in our co-culture model. The glial cell viability was reduced after concentration-dependent incubation with PHE and CBZ, especially in M30 co-cultures (Dambach et al., 2014). Another *in vitro* study focusing on metabolic effects of AEDs showed CBZ-induced stress on primary astrocytes at all concentrations, but low concentrations of GBP did not change the metabolic activities of astrocytes and did not have toxic effects on these cells (Pavone and Cardile, 2003).

Moreover, Haghikia et al. (2008) showed that treatment of inflammatory glia co-culture model with levetiracetam (LEV), an established second-generation AED, reconstituted the impaired astroglial gap junction coupling and membrane resting potential (MRP) to non-inflammatory level. In another study, LEV restored IL-1β-mediated MRP depolarization to physiological levels and promoted anti-inflammatory TGF-β1 expression in inflammatory and control astrocyte-microglia co-cultures (Stienen et al., 2011). LEV and TGF-β1 induced comparable effects on the generation of astrocyte voltage-gated currents in inflammatory co-cultures and the effects of LEV were prevented by antibody to TGF-β1, indicating that the anti-inflammatory effects of LEV on astroglia are mediated *via* TGF-β1 regulation. In addition, LEV suppressed microglial activation including morphological changes, phagocytic activity and cytokine expression in contrast to VPA and CBZ during epileptogenesis (Itoh et al., 2019). Other study findings suggested neuroprotective effects of LEV *via* anti-angiogenesis and anti-inflammatory activities against blood–brain barrier dysfunction in the acute phase of epileptogenesis after status epilepticus (Itoh et al., 2016; Ismail and Faustmann, 2021). In addition, LEV reduced reactive gliosis and expression levels of IL-1β in the hippocampus and the piriform cortex of chronic epileptic rats unlike VPA (Kim et al., 2010).

In summary, these data suggest that astrocyte dysfunction and glia-mediated inflammation play an important role in epilepsy. So, astrocytes and microglia are potential novel targets for alternative anti-epileptogenic therapies.

### Psychotropic Drugs

According to the hypothesis that cytokines may play a role in the pathophysiology of psychiatric disorders (Na et al., 2014; Kim et al., 2016; van den Ameele et al., 2016), further investigations on glia cells offer new findings with regard to CNS inflammation, psychiatric disorders and pharmacological treatment. Venlafaxine, a norepinephrine-serotonin reuptake inhibitor and frequently used drug in mood disorders, revealed anti-inflammatory effects in our astroglia-microglia co-culture model (Table 1). In M30 co-cultures, microglia changed to the RRT, depolarization of membrane resting potential was reversed and an increase of TGF-β level was found in parallel with a reduction of IFN-γ and IL-6 (Vollmar et al., 2008). Corresponding, IL-6 is a major cytokine under pathological conditions in the CNS such as MS, Alzheimer’s disease, trauma, and meningitis (Gruol and Nelson, 1997). Consistent with these findings, venlafaxine was found to be neuroprotective after stroke events in rats (Zepeda et al., 2016) and a decreased microglia staining was described in dorsal root ganglia in a rat model of neuropathic pain (Zychowska et al., 2015). Metabolic profiling of astrocytes treated with venlafaxine revealed effects on amino acids metabolism, cellular growth and proliferation (Sun et al., 2017). In addition, a hyper-ramification of microglia was found in a mice-model of depression and could be reversed by venlafaxine (Hellwig et al., 2016). In a prenatal stress model in Wistar rats, venlafaxine showed protective effects on microglia (Obuchowicz et al., 2020). Further, venlafaxine had inhibitory effects on superoxide generation in LPS-stimulated BV-2 microglia cell line (Dubovický et al., 2014). In conclusion, these findings indicate significant effects of the psychotropic drug venlafaxine on glial cells, underlying additional pharmacological mechanisms.

### Mechanisms of Action of Neurotrophic Drugs on Glial Cells

In recent years, more and more studies focused on mechanisms of action of neurotrophic drugs on glial cells. In our astrocyte-microglia co-culture model of inflammation, the anti-inflammatory properties of the AED LEV on electrophysiological properties of astroglia have been shown to be mediated *via* TGFβ1 regulation (Stienen et al., 2011). Combination of a μ-opioid receptor antagonist at ultralow concentrations and a μ-opioid receptor agonist with LEV managed to activate the G_i/o_ protein and Na^+^/K^+^-ATPase activity, inhibit the G_s_ protein, and decrease the release of IL-1β, contributing to restoration of inflammation-reactive astrocytes (Block et al., 2013). In addition, this combination with LEV downregulated also the glutamate-evoked intracellular Ca^2+^ release and toll-like receptor 4 (TLR4) expression on inflammatory active astrocyte cultures (Hansson et al., 2018). Another study showed combined mechanisms of LEV in rat cortical primary cultured astrocytes including inhibition of AMPA- and adenophostin A (AdA)-induced astroglial release of kynurenine-pathway metabolites, inhibition of IFN-γ-induced inositol 1,4,5-trisphosphate (IP3) receptor activation. Further, LEV reduced the IFN-γ-induced release of cinnabarinic and quinolinic acid, and enhanced the stimulatory effects of IFN-γ on kynurenic acid (Fukuyama and Okada, 2018). Kynurenic acid is known as anti-absence and anti-convulsive metabolite, whereas cinnabarinic acid is a pro-absence and quinolinic acid a pro-convulsive metabolite. Further, it has been demonstrated that LEV inhibits Aβ-induced vesicular glutamate release from human astrocytes (Sanz-Blasco et al., 2016). Another study observed that treatment with LEV stimulated the expression of both brain-derived neurotrophic factor (BDNF) and inducible nitric oxide synthase (iNOS) in a concentration-dependent manner on rat cortical astrocyte cultures, suggesting neuroprotective and anti-inflammatory effects (Cardile et al., 2003). LEV reduced reactive astrogliosis and microgliosis *via* attenuation of IL-1β and IL-1RI expression levels in chronic epileptic rats (Kim et al., 2010). The IL-1β function was linked to inhibition of gap junctions in astrocytes and epileptic activity. These results support multiple anti-inflammatory mechanisms of actions of LEV in neuroglia, especially with regard to epileptic brains. In contrast, VPA did not change the IL-1β and IL-1RI expression levels in astrocytes and microglia (Kim et al., 2010). LEV also attenuated the expression of TNF-α and IL-1β in animal model of status epilepticus. In this model, VPA also inhibited the pro-inflammatory cytokine expression, whereas CBZ did not have effects on cytokines. Moreover, LEV was able to suppress mononuclear phagocyte activation in contrast to VPA and CBZ. The BV-2 microglial activation was also not affected by VPA and CBZ compared to LEV (Itoh et al., 2019). A recent study demonstrated that the number of GABAergic synapses is reduced by VPA-exposed astrocytes, indicating impaired synaptogenesis of inhibitory neurons by VPA-exposed astrocytes (Takeda et al., 2021). It has been discussed that the altered GABAergic synapse formation and synaptic transmission may be caused by a reduced level of protein tyrosine phosphatase receptor type delta (PTPRD), because PTPRD is involved in GABAergic presynaptic differentiation (Takeda et al., 2021). This may indicate an impaired astrocyte-mediated neurodevelopment in case of maternal use of VPA. With regard to treatment with CBZ, upregulated A_1_-receptor mRNA expression in primary astrocyte cultures from brain regions with low receptor expression was detected and linked to phosphoinositol signaling pathway (Biber et al., 1999). Otherwise, CBZ attenuated LPS-induced iNOS expression predominantly by inhibiting phosphatidylinositol 3-kinase (PI-3K)/Akt signaling pathway in activated BV-2 microglial cells (Wang et al., 2014). Acute and chronic treatment of primary cultured astrocytes with CBZ inhibited excitatory astroglial glutamatergic transmission associated with IP3-R and AMPA-R. Further, the pro-inflammatory cytokines IFN-γ and TNF-α induced astroglial L-glutamate release was inhibited by CBZ *via* chronically activation of adenosine A_2A_ receptor, suggesting potential anti-inflammatory effects of CBZ in neuropsychiatric disorders associated with pro-inflammatory cytokines (Okada et al., 2019).

The neuronal mechanism of action of the antidepressant venlafaxine is believed to be mediated by uptake inhibition of norepinephrine and serotonin. Serotonin uptake is already inhibited at low doses, whereas at high doses norepinephrine and serotonin uptakes are inhibited (Harvey et al., 2000). Astrocytes express norepinephrine transporters and could inactivate norepinephrine that escapes neuronal re-uptake. This effect seems to be inactivated by antidepressants such as venlafaxine (Inazu et al., 2003). On intracellular level, the selective serotonin and serotonin norepinephrine reuptake inhibitors (SSRI and SNRI) decreased TNF-α and nitric oxide production in microglia. This mechanism is suggested to be cAMP mediated, suggesting that cAMP signaling is involved in regulation of the anti-inflammatory response. This effect was induced more by fluoxetine and paroxetine compared to venlafaxine (Tynan et al., 2012). Further, venlafaxine regulated inflammation in astrocytes by inhibition of JNK1 activity and STAT3 basal activity, which reduces the production of IL-6 and IL-1β. This effect on STAT3 was independently from a previous induction by a cytokine mixture (comprising complement component 1q, TNF-α, IL-1α). Venlafaxine here revealed a low cytotoxicity on astrocytes compared to other antidepressants (He et al., 2021). Additionally, glioma cells pretreated with venlafaxine and isoproterenol revealed an increased p90Rsk phosphorylation compared to isoproterenol alone, indicating further intracellular effects of venlafaxine on neuro-glial pathways (Khawaja et al., 2004).

In summary, future studies are necessary to reveal further mechanisms of action of neurotrophic drugs with regard to glial cells, because not only neuronal modification and excitability are crucial, but also aspects of glia-mediated pathomechanisms, especially glia-induced inflammation.

### Immunomodulatory Drugs

A link between inflammation and glial cells in inflammatory CNS diseases, e.g., autoimmune diseases such as MS or infection diseases such as meningitis suggested a potential regulatory effect of dexamethasone and IFN-β on glial cells. In our glial co-culture model of inflammation, dexamethasone reversed an LPS-induced microglial activation, compromised astroglia membrane potential, cellular coupling and Cx43 expression (Hinkerohe et al., 2010), indicating anti-inflammatory and regulatory effects on glial network (Table 1). Consistent with these findings, dexamethasone reduced neuroinflammatory response and migration of LPS-activated microglia BV2 cells (Hui et al., 2020). Interestingly, Park et al., 2019 showed that dexamethasone induces a specific form of ramified microglia with missing microglia signature genes, e.g., TMEM119 or P2RY12, leading to the conclusion of a dexamethasone-induced dysfunctional microglia type (Park et al., 2019). Further, β2-adrenoreceptor-mediated inflammation can be reduced by dexamethasone in astrocytes and microglia (Ryan et al., 2020). Dexamethasone significantly reduced seizure-induced microglia activation in rats and could reduce the long-term effects of first-time seizures (Fox et al., 2020). In an experimental autoimmune encephalomyelitis model, dexamethasone delayed the inflammatory activation of microglia and astrocytes in the white matter of spinal cord (Nam et al., 2021). In our co-culture model, pre-incubation with IFN-β prevented microglial activation in the physiological M5 co-culture despite use of TNF-α, IL-1β, and IFN-γ as activators (Hinkerohe et al., 2005). In addition, IFN-β mediated the release of anti-inflammatory IL-10 from microglia in mice (Lobo-Silva et al., 2017) and reduced the number of reactivated microglia in a retinal model (Behnke and Langmann, 2020).

In terms of connectivity, rapidly proliferating astrocytes exposed to dexamethasone failed to express any assemblies (Landis et al., 1991). Interestingly, dexamethasone increased the gap junctional intercellular communication in our M30 co-cultures and reduced it in rat and human glioma cell lines (Hinkerohe et al., 2011). Further, the hetero-cellular gap-junctional coupling between F98 glioma cells and glia cells was reduced by dexamethasone in our co-culture model (Ismail et al., 2017).

Taken together, several studies strongly indicate regulatory effects on inflammatory activation, cytokine release and functional coupling by dexamethasone and IFN-β in glial networks.

### Others

Hepatic encephalopathy (HE) is a neuropathological condition caused by acute or chronic liver failure due to hyperammonemia and impaired detoxification of ammonia by the astrocytic glutamine synthetase, resulting in astrocyte swelling (Brusilow et al., 2010). It is known that microglia may contribute to the astrocyte swelling induced by ammonia (Rao et al., 2013). In our M30 co-culture model, ammonia reduced the glia-cell viability (Ismail et al., 2021). Furthermore, microglial activation was detected after incubation with ammonia under physiological and pathological conditions (Table 1). Ammonia effects on Cx43 and aquaporin 4 expression were limited (Ismail et al., 2021). The microglial activation was consistent with previously described findings of up-regulated microglia activation marker ionized calcium-binding adaptor molecule-1 (Iba-1) in post-mortem brain tissues (from HE patients) and cultured microglia (treated with ammonia) (Zemtsova et al., 2011). In another study, ammonia attenuated LPS-induced microglia reactivity including upregulation of pro-inflammatory cytokines in an astrocyte-dependent way (Karababa et al., 2017). Studies about effects of ammonia on astrocytes and microglia contribute to better understanding of pathophysiological mechanisms of HE.

## Conclusion

The *in vitro* astrocyte-microglia co-culture model of inflammation developed by Faustmann et al. (2003) allowed to study the endogenous inflammatory reaction and the cytokine expression under drugs in a differentiated manner. In addition to astrocytes, investigations on microglia offer a whole network approach leading to better understanding of non-neuronal cells and their pathological role in CNS diseases and treatment. Effects of commonly used AEDs (e.g., LEV, VPA, CBZ, GBP, and PHE), immunomodulatory drugs (e.g., dexamethasone, IFN-β) and psychotropic drugs (e.g., venlafaxine) have been already demonstrated, contributing to better understanding mechanisms of actions of CNS drugs and their pro- or anti-inflammatory properties concerning glial cells. Furthermore, influence of drugs on glial cell viability, proliferation and astrocytic network has been shown. The *in vitro* astrocyte-microglia co-culture model of inflammation proved to be suitable as unique *in vitro* model for pharmacological investigations on astrocytes and microglia with future potential (e.g., cancer drugs, antidementia drugs, and toxicologic studies). Even more, astrocytes and microglia as main glia cells are novel therapeutic targets for future treatment perspectives using the glia co-culture model of inflammation.

## Author Contributions

FSI, FC, and TJF prepared the manuscript. All authors were responsible for concepts and design, contributed intellectually, acquired, analyzed, and interpreted the data, read and approved the submission, and agreed to be accountable for the content of the work.

## Conflict of Interest

The authors declare that the research was conducted in the absence of any commercial or financial relationships that could be construed as a potential conflict of interest.

## Publisher’s Note

All claims expressed in this article are solely those of the authors and do not necessarily represent those of their affiliated organizations, or those of the publisher, the editors and the reviewers. Any product that may be evaluated in this article, or claim that may be made by its manufacturer, is not guaranteed or endorsed by the publisher.
